# The Role of Establishing a Multidisciplinary Team for Idiopathic Granulomatous Mastitis in Improving Patient Outcomes and Spreading Awareness about Recent Disease Trends

**DOI:** 10.1155/2020/5243958

**Published:** 2020-01-27

**Authors:** Rami J. Yaghan, Nehad M. Ayoub, Shadi Hamouri, Alia Al-Mohtaseb, Maha Gharaibeh, Lamees Yaghan, Mahmoud Al-Dari, Hiba Al-Kaff, Nabil A. Al-Zoubi

**Affiliations:** ^1^Department of Surgery, College of Medicine and Medical Sciences, Arabian Gulf University, Manama, Bahrain; ^2^Department of Surgery and Urology, Faculty of Medicine, Jordan University of Science and Technology, Irbid, Jordan; ^3^Department of Clinical Pharmacy, Faculty of Pharmacy, Jordan University of Science and Technology, Irbid, Jordan; ^4^Department of Pathology and Microbiology, Faculty of Medicine, Jordan University of Science and Technology, Irbid, Jordan; ^5^Department of Diagnostic Radiology and Nuclear Medicine, Faculty of Medicine, Jordan University of Science and Technology, Irbid, Jordan

## Abstract

**Background:**

Iidiopathic granulomatous mastitis (IGM) is stereotypically described as a mysterious entity that mimics breast carcinoma imposing management challenges. In 2002, we established a multidisciplinary team to treat patients with IGM. This study aimed to evaluate the role of this team in improving patient outcomes. Also, a review of literature is provided to highlight recent disease trends. *Patients and Methods*. Pertinent data for 44 patients treated for IGM from 2002 to 2018 were analyzed and compared to data prior to 2002.

**Results:**

Mean age at diagnosis was 37.9 years ± 6.4. The diagnosis of IGM was confirmed by True-cut biopsy (TCB), Frozen section (FS), and surgical biopsy in 70.5%, 25%, and 4.5% of patients, respectively. FS was used to assess the resection margins in three patients. Suspicion for malignancy was raised in one out of 39 ultrasound reports, and one out of 20 mammography reports. Wide local excision was the main treatment modality (95.5%). 19 patients (43.2%) received corticosteroids. Prior to 2002, IGM was only recognized after surgical resection with a 71% initial false impression of carcinoma. After 2002, the initial false clinical impression of carcinoma dropped to 29.5%. Recurrence rate was 31.82%. Younger age at diagnosis was significantly associated with recurrence (*χ*^2^ = 5.598; *p* = 0.018). Chi-square analysis showed no significant association between BMI and recurrence (*χ*^2^ = 0.776; *p* = 0.678).

**Conclusion:**

The establishment of a multidisciplinary team for IGM was associated with a reduced erroneous impression of breast cancer, and a reduced false positive radiological diagnosis of breast carcinoma. FS was a useful confirmatory procedure. Our series included the first case of a diffuse papular rash as a systemic manifestation of IGM. Recent literature indicates that IGM is changing its face. IGM is being reported in all age groups, and even in males. The clinical manifestations have markedly expanded. Diagnosis by TCB has replaced blind surgical excision. More data regarding predictors of recurrence is accumulating.

## 1. Introduction

The term granulomatous mastitis (GM) describes two main entities: idiopathic granulomatous mastitis (IGM) and secondary (specific) GM. IGM is a rare disease of the breast. It was recognized as a different entity from other types of mastitis in 1972 [1]. Histologically, IGM is characterized by the presence of chronic noncaseating granulomas centered on breast lobules [1, 2]. IGM is a diagnosis of exclusion. Careful histopathological review of biopsy specimens, as well as microbiological analyses are essential to rule out secondary GM.

Since its first description 47 years ago [1], IGM is still stereotypically being described as a mysterious entity that mimics breast carcinoma clinically, radiologically, and even cytologically [3]. The classical patient presents with a hard breast lump [4]. Ultra-sonographic and mammographic findings are usually nonspecific and are occasionally interpreted as malignant [5, 6]. This frequently leads to an initial misdiagnosis as breast carcinoma [7].

The etiology of IGM is unknown. The most widely adopted theory considers IGM to be a local immune response that involves both humoral and cell-mediated immunity, and results in noncaseating granulomas [8]. The triggering antigen could be an unidentified infectious agent, external antigen, or a glandular secretory antigen [6]. Associations with parity, lactation, and pregnancy are universal findings [3, 4, 6]. Hyperprolactinemia and *α*1-antitrypsin deficiency, have been reported in some patients with IGM [3].

Surgical excision and systemic immunosuppressive agents are the main treatment modalities [4, 7, 9, 10]. However, no universal guidelines exist regarding when to treat by surgery, corticosteroids, or both leaving the decision for the treating surgeon or physician preference. IGM is associated with a high rate of local recurrence, exceeding 15% in most studies [7, 9, 11]. Identification of the risk factors associated with recurrence of IGM is currently a major concern. Modification of treatment modalities according to the presence or absence of these factors is another issue to be addressed.

Being a rare disease, all available data regarding risk factors, treatment modalities, and predictors of recurrence of IGM depended on case reports, or retrospective case series. This imposed understandable limitations for the applicability of regression analyses [12]. Multicenter and meta-analysis studies are mandatory to obtain statistically sound data. Only a limited number of such studies have been published recently, but still with a limited number of variables [9, 11, 13].

In summary, the rarity of IGM, its clinical resemblance to breast carcinoma, absence of specific radiological findings, unknown etiology, lack of universal treatment guidelines, and high rate of local recurrence contribute to the classical stereotypic description of IGM as a mysterious entity that is difficult to diagnose and treat. In particular, the similarity of the clinical presentation of IGM to breast carcinoma complicates the diagnosis and creates temporary patient fear and anxiety. It may also delay the initiation of systemic treatment.

In 2002, a multidisciplinary team was established in our center to treat patients with IGM. The team included a surgeon, a physician, a radiologist, a pathologist, and a clinical pharmacist. All patients with the diagnosis of IGM or a suspected diagnosis of IGM are referred to this team. This study aimed to evaluate the impact of awareness about IGM raised by the establishment of this team in modifying the management of IGM. In particular, the effect on the rate of an initial erroneous impression of carcinoma, the changing trends in confirming the diagnosis, and the changing trends in the treatment will be highlighted. We will also review the recent literature critically to highlight the current face of IGM.

## 2. Patients and Methods

Patients treated for IGM at King Abdulla University Hospital (KAUH) between January 2002 and December 2018 were the subject of this study. The institutional review board approved the research (registration number 11/101/2016). All the procedures used for data collection complied with the Jordanian research regulations and the Helsinki declaration. An informed written consent was obtained from each patient before commencing treatment. We reviewed the literature to monitor any changing trends of the disease.

### 2.1. Inclusion and Exclusion Criteria

Female patients with proven histological diagnosis of IGM were included in this study. This necessitated the availability of a pathology report consistent with the diagnosis of IGM. We adopted the classical description of IGM first published by Kessler and Wolloch [1], and Cohen [2]. In every case, the characteristic noncaseating granulomatous lesions diffusely obliterating the lobular structure (with presence of epithelioid histiocytes, lymphocytes, plasma cells, polymorphonuclear leukocytes, and multinucleated Langhans-type giant cells) constituted the primary diagnostic feature. Cases of the secondary GM associated with tuberculosis, duct-ectasia, fat necrosis, post-surgical foreign body reactions, fungal infection, and sarcoidosis were excluded. Ziehl–Neelsen stains for acid-fast bacilli and periodic acid-Schiff reactions were negative. A single pathologist (AA) who was blind to patient demographic and clinical data reviewed the histopathological slides for the sake of this study to confirm the diagnosis of IGM.

### 2.2. Data Collection

Pertinent data regarding patient demographics, clinical presentation, investigations, treatment, and recurrence were retrieved from the electronic database at KAUH, and from patient files. KAUH is the major tertiary referral center for the North of Jordan and is affiliated to the Faculty of Medicine at the Jordan University of Science and Technology. The initial rate of false clinical suspicion of carcinoma and month at presentation were recorded.

### 2.3. Statistical Analysis

Data analysis was performed using SPSS version 21.0 (IBM SPSS, Armonk, NY, USA). Continuous variables were presented as mean ± standard deviation. Categorical variables were presented as the frequency and percentages. Pearson's chi-square (*χ*^2^) test of independence was used to assess associations between categorical variables. To assess correlations between continuous variables, Spearman's correlation test was applied. All *p*-values were two-sided and *p* < 0.05 was considered to indicate a statistically significant difference.

Whenever possible, we compared the data in this report to previous published data from our center [14], in order to explore the influence of awareness by our clinicians on the management of IGM after the year 2002.

## 3. Results

**Figure 1:**
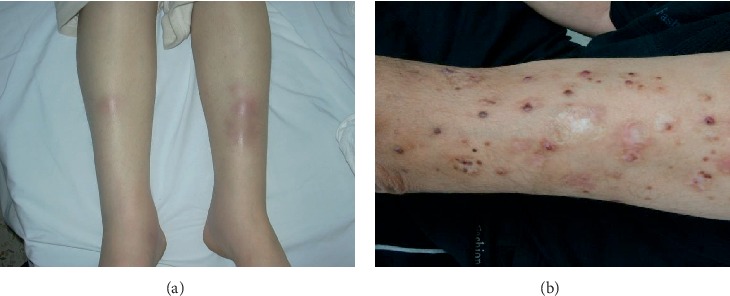
Examples of extramammary manifestations of Idiopathic granulomatous mastitis (IGM). (a) Erythema nodosum. (b) Papular rash: this 30-year-old patient presented with a hard breast mass and diffuse papular rash with pustules in both lower limbs. True-cut biopsy of the breast mass revealed IGM. The skin lesions disappeared 7 days after wide local excision of the beast lesion and the commencement of prednisolone treatment (patient had old burn scars).

Initially we identified 85 patients with the diagnosis of GM. After applying the above inclusion and exclusion criteria, 40 patients were excluded because they proved to have secondary GM. Another male patient was excluded from the study. The remaining 44 female patients having IGM were the subject of this study. The general features and clinical characteristics of the patients are summarized in Table 1. The mean age at diagnosis was 37.9 years ± 6.4 (range 25–52). More than half of the patients were overweight or obese at presentation and the vast majority were parous (97.7%). An identifiable breast mass was present in all patients but with variable additional local and systemic features (Table 1). Four patients (9.1%) had extramammary manifestations. Two of the three patients who were having erythema nodosum (EN) were pregnant at the time of diagnosis (Figure 1). Among patients who had extramammary manifestations, one patient presented with papular rash and pustule formation in her lower limbs (Figure 1). These lesions disappeared completely by the seventh day after excising the breast mass and starting prednisolone treatment.

The diagnosis of IGM was confirmed by True-cut biopsies (TCBs) in 31 patients (70.5%), per-operative frozen sections (FS) in 11 patients (25%), and excisional biopsies in 2 patients. Initially, five patients with proven IGM by TCBs had FS to judge the appropriateness of FS in confirming the diagnosis of IGM. The FS results in these five patients matched the TCB results. Thereafter, in 11 patients, the histological diagnosis was based solely on FS. The permanent histological studies were consistent with the FS diagnosis of IGM. All possible causes of secondary GM were ruled out before issuing the final permanent histological report. In these 11 patients, the average time from seeing the patient in the clinic to having definitive surgery was 2.1 days (range: 1–4 days), with an average extra operative time of 32.55 minutes (range: 29–40 minutes). In three patients, FS was also used to evaluate the adequacy of resection margins. Both TCB and FS provided a 100% accuracy rate in confirming the diagnosis of IGM. Fine needle aspiration cytology (FNAC) was performed for 14 patients (31.8%); the results were consistent with a benign lesion in 8 patients, diagnostic of a granulomatous infection in one patient, and suspicious for malignancy in 5 patients.

Ultrasonography was performed for 39 patients and mammography for 20 patients. A high suspicion of malignancy was raised in one ultrasound report, and in one mammography report. Treatment consisted of a wide local excision in 42 patients, and mastectomy in one patient. The remaining patient was pregnant and refused any surgical or medical treatment, with no further follow up. The single patient who had mastectomy was having a destructive form of IGM. Mastectomy was the only surgical option. Prednisolone was given to 19 (43.2%) patients. Doses and durations of prednisolone treatment were individualized according to the needs of each patient as judged by the treating team. The doses varied from 10 mg twice daily to 30 mg twice daily. The duration varied from one to three months.

**Figure 2:**
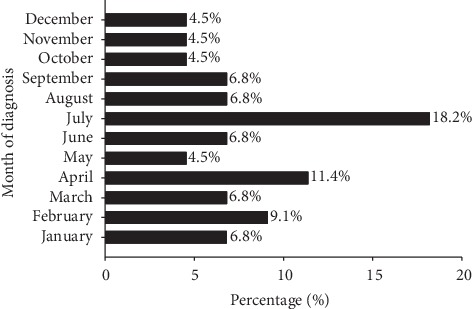
Month at presentation of IGM. There was a trend toward an increased diagnosis in the warm seasons (spring and summer).

Twenty-seven patients (61.4%) presented during the summer and spring seasons (Figure 2).

### 3.1. Recurrence of IGM

Data related to the recurrence of IGM are summarized in Table 2.

Fourteen patients out of 44 patients (31.8%) developed recurrent IGM. Majority of recurrences were ipsilateral (71.4%) (Table 2). Average number of recurrent episodes was 1.7 (range 1–4). Ten of the 14 patients (71.4%) who developed recurrence presented initially during the spring and summer seasons.

One of our patients had hyperprolactinemia due to a pituitary tumor; she was treated by a combination of surgery and prednisolone. She had 4 recurrences of IGM until her adenoma was controlled by surgery.

Association analysis for categorical variables indicated that age at diagnosis was significantly associated with disease recurrence among IGM patients (*χ*^2^ = 5.598; *p* = 0.018). Forty-eight percent of patients who developed recurrent disease were at the age range of 20–40 years compared to 8.4% of patients who had recurrence when diagnosed at age greater than 40 years. In line with this, 91.7% of patients who were diagnosed at age above 40 years reported no disease recurrence compared to 52% of patients who were diagnosed at age less than 40 and did not have recurrence. However, age was not significantly associated with side or site of the disease, as well as presence of pain, skin redness, lymphadenopathy, and the need for prednisolone treatment [data not shown]. Interestingly, the age at diagnosis was significantly associated with the presence of nipple retraction (*χ*^2^ = 8.235; *p* = 0.004). None of the patients diagnosed with IGM at the age of 20–40 years presented with nipple retraction. Of patients diagnosed at age more than 40 years, half presented with nipple retraction. Chi-square analysis for associations between BMI group and recurrence status showed no significant association (*χ*^2^ = 0.776; *p* = 0.678). In addition, obesity was not associated with disease presentation in terms of side of the disease, site, skin redness, nipple retraction, lymphadenopathy, and the need for prednisolone treatment [data not shown].

The correlations between clinical characteristics for patients diagnosed with IGM with age and BMI at presentation are shown in Table 3. There was an inverse significant correlation between BMI and size of the mass in patients diagnosed with IGM (rho = −0.570, *p* = 0.007, Table 3). Among patients with recurrent disease, age at diagnosis was significantly correlated with time to recurrence (rho = −0.836, *p* = 0.003, Table 3). This correlation is inverse indicating that older patients tended to have shorter time for disease recurrence. BMI was also inversely and significantly correlated with the size of the mass in patients who had recurrence (Table 3).

Diagnostic modalities, treatment modalities, and rates of clinical diagnosis for IGM at KAUH before and after the year 2002 are summarized in Table 4.

## 4. Discussion

### 4.1. Clinical Presentation

The first ten reported cases of IGM by Kessler and Wolloch, and Cohen shared the classical presentation of a hard cancer-mimicking breast mass occurring in young parous women [1, 2]. However, the spectrum of possible local presentations of IGM has expanded to include acute breast-abscess like presentations, and the subacute presentations with skin fungation, fistulization, and/or sinus formation [6, 7, 15]. Extramammary presentations in the form of EN, arthralgias, arthritis, oligo-arthritis, and episcleritis are recognized occasional findings [3, 16, 17]. One of our cases of IGM presented with associated papular rash in the lower limbs (Figure 1). These lesions disappeared after excising the breast mass and starting the patient on prednisolone. No similar extramammary skin manifestation has been reported previously. As was the case in our group of patients, the vast majority of IGM cases are reported in young women in association with parity and lactation [3, 4, 6]. However, the age spectrum has expanded to include postmenopausal women, as well as prepubertal girls [14, 18]. A very limited number of IGM cases have also been reported in males [7, 19]. Among our study group all the above mentioned variant presentations were encountered (Table 1).

The expanding patterns of the clinical presentation of IGM further complicate the treatment options.

At our center, prior to the year 2002, the diagnosis of IGM was only recognized after surgery, with an initial clinical impression of breast carcinoma in 71% of the cases [14]. In sharp contrast to this, after the year 2002, the possible diagnosis of IGM was considered at the initial presentation in 31 cases (70.5%) (Table 4). This reflects the impact of awareness about IGM by our clinicians resulting from the establishment of a special multidisciplinary team for the management of IGM after the year 2002. The presence of a sizable hard breast mass of a relatively short duration of onset, the presence of associated variable degrees of local breast pain and inflammation, and a high index of suspicion increase the likelihood of early clinical diagnosis of IGM.

### 4.2. Radiological Investigations

IGM usually produces nonspecific ultra-sonographic and mammographic findings, which occasionally can be interpreted as malignant [20]. Mammographic findings in IGM include focal or regional asymmetry, a solitary mass or masses, skin thickening, skin and nipple retraction, and axillary lymphadenopathy [6]. The most common ultrasound finding is a hypoechoic or heterogeneous mass or masses, with characteristic tubular hypoechoic extensions [6]. Doppler imaging demonstrates increased internal blood flow within the lesions and surrounding parenchyma [6].

Investigators are evaluating the value of new techniques in increasing the specificity of ultrasound in diagnosing IGM. In this context, Arslan et al. showed that the combination of strain elastography and B-mode ultrasound was superior to B-mode ultrasonography alone in differentiating IGM from malignant breast lesions [21]. Teke et al. evaluated the role of acoustic radiation force impulse imaging in differentiating IGM from breast cancer [20]. They showed that the addition of virtual touch tissue imaging and virtual touch quantification as a complement to conventional ultrasound provide viscoelastic properties of tissues, that increase the specificity of ultrasound [20]. However, such techniques are available in few centers only limiting their applicability.

Among our study group, a false suspicion of breast carcinoma was encountered in only one mammographic report and one ultra-sonographic report. This reflects the positive impact of awareness about IGM by our radiologists as part of the multidisciplinary team. Direct contact between the clinician and the radiologist is of at most importance in improving the radiological diagnostic accuracy of IGM.

Several studies have evaluated the role of MRI in differentiating IGM from breast neoplasms. The general trend is that MRI is not very helpful in differentiating GM from malignancy. Both lesions share identical features on MRI [6, 22]. A specific but rare MRI finding in IGM is a peripherally enhancing cystic or solid mass with fistulous tract to the skin [23]. At our center, based on a previous limited experience [24], MRI is not part of the diagnostic work up for IGM. However, MRI might be of value in assessing the response to treatment in some cases [6].

### 4.3. Confirmatory Diagnostic Tools

Traditionally IGM was a surprising histological diagnosis after surgical resection [14]. Currently, most of the reported cases of IGM are diagnosed by TCBs [8, 11]. TCBs provide a definitive diagnosis of IGM, and allow for a wider use of preoperative steroid treatment [25]. Among our study group, the diagnosis of IGM was confirmed by TCBs in 31 patients (70.5%), FS in 11 patients (25%), and excisional biopsies in 2 patients. We introduced the use of FS in confirming the diagnosis of IGM. Our results suggest that FS can be a time-saving procedure when the clinical diagnosis of IGM is raised. In addition, we found that FS was useful in assessing the resection margins of IGM. Gross identification of free resection margins during surgical resection of IGM is sometimes difficult, especially if severe inflammation and pus are present [15]. No previous reports exist in regard to using FS in IGM.

Cytological diagnosis of GM is challenging [26]. FNAC in IGM might show some atypia. There have been scattered reports about patients with IGM undergoing unnecessary mastectomies, because of a misleading diagnosis of malignancy based on clinical, radiological, and FNAC results [14, 27]. Among our study group, FNAC was misleading. No major decisions are based solely on FNAC at our breast unit especially in young women.

### 4.4. Etiology

Etiology of IGM is unknown. The most widely adopted theory considers IGM to be an immune response that involves both humoral and cell-mediated immunity [8]. The nature of extramammary manifestations of IGM, including inflammatory arthritis, arthralgias, episcleritis, and EN, is suggestive of an underlying immune process [6, 17]. A favorable response to corticosteroids is in harmony with this pathogenesis [10, 16, 25]. However, serological tests that are usually positive in patients with autoimmune diseases (such as rheumatoid factor and antinuclear antibody) have not demonstrated any consistent diagnostic or prognostic value in IGM [3].

Cystic neutrophilic GM (CNGM) is a recently identified entity that stimulated the reconsideration of an underlying infectious bacterial etiology for IGM [28–30]. The typical biopsy in CNGM reveals the presence of granulomatous inflammation with characteristic cystic spaces lined by neutrophils containing Gram-positive cocci [31]. *Corynebacteria*, especially *Corynebacterium kroppenstedtii*, and to a lesser extent *Staphylococcus* spp. were detected in some patients with CNGM by culture, or by 16S rRNA gene sequencing of specimens obtained at surgery or FNAC [29, 31]. A minority of cases diagnosed previously as IGM might turn to be CNGM. Kıvılcım et al. evaluated multiple bacteriologic agents that might play a role in the etiology of IGM using a nucleic-acid-based assay with a universal primer on previously obtained IGM biopsies without evidence of CNGM [32]. They obtained no positive results [32].

In brief, when the characteristic histological findings of CNGM are encountered, the pathologist should make every effort to look for an associated primary bacterial infection. Culture of fresh specimens, Gram staining, or rRNA gene sequencing of specimens obtained at surgery might be considered in these cases [29, 30]. CNGM is now considered a secondary form of GM [28, 29]. Early recognition of CNGM might call for a first line of treatment by lipophilic antimicrobials [28]. A multidisciplinary team advice should be sought. Our series did not include any case with histological suggestion of CNGM.

Among our study group, we observed a trend toward an increased occurrence of IGM during the warm seasons. No statistical evidence could be drawn in this regard in view of the small number of cases (Figure 1). We did not encounter any data in the literature in this regard. If such a trend is observed in further studies, it will be in favor of an infectious or a seasonal antigen, as a triggering agent for IGM.

### 4.5. Treatment, Recurrence, and Classification of IGM

Many questions are raised when treating a patient with IGM: Shall we treat by surgery, corticosteroids, or both? If both, shall we start by surgery or corticosteroids? What is the optimal dose and duration of corticosteroid therapy? What is the reflection of a given treatment on the likelihood of recurrence [9]? Does the presence of an extramammary manifestation alter the treatment plan?

Currently there are no clear-cut answers for these questions. However, some clues can be extrapolated from recent literature. In any institution, the treatment decision should be the responsibility of a multidisciplinary team. Individual patient needs should be taken into consideration.

Wide local excision has stood the test of time as being a corner stone in the treatment of IGM [7, 9, 14]. In a recent systematic review and meta-analysis surgical treatment (with or without steroids) was associated with a high cure rate and a relatively low recurrence rate [13]. Mastectomy with breast reconstruction is reserved for severe destructive cases of IGM [33], as was the case in one of our patients.

A growing number of publications over the last two decades have also shown the effectiveness of corticosteroid treatment in reducing the extent of surgery, or even alleviating the need for surgery in selected cases [4, 7, 11]. Administration of steroids for large lesions prior to surgery may help in obtaining better cosmesis [34]. On the other hand, the use of corticosteroids might be limited in pregnant, diabetic, or lactating women. Many women will be reluctant to receive steroids in view of the potential adverse events.

Among our study group, wide local excision was the sole treatment for IGM in the absence of local signs of inflammation and extramammary manifestations. Steroids were administered if local signs of inflammation or extramammary manifestations were present. Our trend toward a wider use of systemic immunotherapy when significant local inflammation is present is supported by recent literature [9, 11, 15, 34]. Also, there is accumulating evidence that the use of systemic immunotherapy is indicated when EN is present [16]. The four patients who were having skin manifestations among our study group received prednisolone with quick disappearance of their systemic manifestations.

Prior to the year 2002 only 16.7% of the patients treated at our center received prednisolone therapy, and those were patients who developed recurrence of IGM (Table 4). The proportion of patients who received prednisolone increased to 43.2% in the current study. This reflects the impact of awareness about IGM by our clinicians.

Gunduz et al. evaluated the role of topical corticosteroids in 11 patients with IGM [35]. He reported a good response in both the skin and parenchyma of the breast. Further studies are needed in this regard. Scattered reports suggested that methotrexate [36], and azathioprine (in combination with steroids) [25] may be other useful second line immunosuppressive agents. Postolova et al. have recently reported their experience in using methotrexate as a second line monotherapy in 19 patients with IGM who failed on previous treatment by antibiotics, surgery, or steroid treatment [37]. Methotrexate was administered orally at a dose of 10–15 mg/week, and increased up to 20–25 mg/week, or shifted to a subcutaneous rout according to the clinical progress. By 15 months of treatment, 94% of their patients demonstrated notable improvement and 75% had disease remission. They concluded that, within the limitation of the small number of patients included in their study and the retrospective nature of their data, methotrexate monotherapy was an effective treatment for IGM [37]. Antibiotics are usually used for secondary infections in IGM. However, a single report suggested the usefulness of rifampicin as a sole treatment for IGM [38].

IGM recurrence rate among our study group was 31.8%. Our data showed that a younger age at diagnosis was significantly associated with a higher disease recurrence (*χ*^2 ^= 5.598; *p* = 0.018). There was a trend for increased recurrence rate of IGM among obese patients, and patients with comorbidity (Table 2). In addition, the presence of pus and severe inflammation were more prevalent in this group (71.4%). Our results showed that IGM patients should be followed for a minimum period of one year. The recurrence might occur in the contralateral side in about one third of the cases (Table 2). At our center, recurrent IGM is treated by reexcision followed by oral prednisolone for a period judged by the treating team.

More data is accumulating regarding the risk of recurrence of IGM. Uysal et al. found a statistically significant association between IGM recurrence and history of pregnancy, breastfeeding, and breast infection; however, comorbidity and obesity were not [9]. Yılmaz et al. found that the number of births, duration of lactation, BMI, presence of fistulas, abscess formation, and luminal inflammation on histological examination were significantly more prevalent in patients with recurrent IGM [12]. They suggested an interesting scoring system for prediction of recurrence of IGM, but the small number of patients was an understandable limit in this regard.

In a previous report, we proposed a clinically based classification for IGM that serves as a prognostic value [15]. We suggested that IGM could be classified into 4 distinct patterns according to the presenting clinical picture. Patterns presenting with marked local inflammation and skin ulceration or fistulization were more likely to develop recurrence.

A comprehensive clinical, radiological, and pathological classification system that provides clues for treatment and predicts the likelihood of recurrence is still lacking.

### 4.6. Weaknesses and Strengths of the Study

Our study is of retrospective nature. The number of cases is not adequate to obtain regression analysis for all potential variables. These weaknesses are shared by other reports on IGM. On the other hand, our results reflect the benefits of establishing a dedicated multidisciplinary team for the management of IGM.

Our review of the recent literature showed that IGM is departing from its stereotypic description. In summary, IGM is being reported in all age groups and in both sexes [7, 18, 19]. The spectra of local and systemic manifestations have expanded markedly [15–17]. Preoperative diagnosis by TCB have replaced blind surgical excision [11]. Modified breast ultrasound techniques are being tested to improve their diagnostic specificity [20, 21]. Evidence is accumulating in favor of using a combination of surgical and immunosuppressive treatment under the care of a multidisciplinary team. The presence of marked local and systemic manifestations calls for a more intensive treatment [6, 9, 15]. Etiology of IGM, however, continues to be an enigma!

## 5. Conclusion

The establishment of a multidisciplinary team for the management of IGM was associated with clinical benefits: the rate of erroneous initial clinical impression of breast cancer was markedly reduced, more patients received corticosteroid treatment, and the false positive radiological diagnosis of breast carcinoma was reduced. We introduced FS as a useful diagnostic tool in IGM that might also help in assessing the resection margins. Our series included the first case of a diffuse papular rash as a systemic manifestation of IGM. Our review of the recent literature showed that IGM is departing from its stereotypic description.

## Figures and Tables

**Table 1:** The general characteristics of 44 patients diagnosed with IGM between 2002 and 2018.

Characteristics	*n* (%)	Notes
*BMI, kg/m^2^*		
Obese	16 (36.36%)	
Overweight	14 (31.82%)	
Normal or underweight	4 (9.09%)	
Missing	10 (22.73%)	
Associated comorbidity	13 (29.55%)	
Diabetes mellitus	4 (9.09%)	
Thyroid disorders	3 (6.82%)	
Hypertension	3 (6.82%)	
Hyperprolactinemia	2 (4.55%)	
Atopic dermatitis	1 (2.27%)	
Missing	5 (11.36%)	
*Menstrual status*		
Menstruating	44 (100%)	
*Parity*		
Parous	43 (97.73%)	37 patients (84.09%) had at least 3 previous pregnancies
Nulliparous	1 (2.27%)	
Pregnancy at presentation	5 (11.36%)	
Lactation at presentation	5 (11.36%)	
Presence of mass	44 (100%)	Mean size: 5.8 cm (range: 1–15)
		Mean duration: 3.01 months (range 0.25–18)
Presence of pain	38 (86.36%)	(Variable from mild to severe)
Abscess-like presentation	16 (36.36%)	
Axillary lymphadenopathy	13 (29.55%)	
Nipple retraction	3 (6.82%)	
Ulcer, sinus, or fistula formation	3 (6.82%)	
*Extramammary findings*		
Erythema nodosum	3 (6.82%)	
Papular rash	1 (2.27%)	
*Side*		
Left	23 (52.27%)	
Right	19 (43.18%)	
Bilateral	1 (2.27%)	
Not recorded	1 (2.27%)	
*Site*		
Upper lateral quadrant	20 (45.45%)	
Retro-areolar	15 (34.09%)	
Upper medial quadrant	2 (4.55%)	
Lower medial quadrant	2 (4.55%)	
Not recorded	5 (11.36%)	
*Surgery*		
Wide local excision	42 (95.45%)	
Mastectomy	1 (2.27%)	
Prednisolone treatment	19 (43.18%)	
IGM suspected clinically	31 (70.45%)	
Clinical suspicion of cancer	13 (29.54%)	

Data presented as frequency and percentage. BMI, Body mass index. BMI was calculated using the standard method as weight in kilograms divided by the square of height in meters. Based on the World Health Organization (WHO) classification for obesity, patients were defined as underweight (BMI < 18.5 kg/m^2^), normal (BMI 18.5–24.99 kg/m^2^), overweight (BMI ≥ 25.00 kg/m^2^), and obese (BMI ≥ 30.00 kg/m^2^) [World Health Organization (WHO), obesity: preventing and managing the global epidemic, WHO Technical Report Series 894, Geneva, Switzerland, 2000].

**Table 2:** The general characteristics of patients developing recurrent IGM (*N* = 14).

Characteristics	*n* (%)
Number of patients	14 (31.82%)
*BMI, kg/m^2^*	
Obese	8 (57.14%)
Overweight	5 (35.71%)
Normal or underweight	1 (7.14%)
Lactation at recurrence	1 (7.14%)
Associated comorbidity	6 (42.85%)
Mean size of recurrent mass	4.44 cm (range: 2–10)
Mean size of original mass	5.57 cm (range: 2–8)
Mean number of recurrences	1.70 (range: 1–4)
Mean time to recurrence	10.64 months (range: 1–72)
Presence of pain	10 (71.43%)
*Side of recurrence*	
Ipsilateral	10 (71.43%)
Contralateral	4 (28.57%)
Abscess-like presentation	7 (50.00%)
Presence of pus at initial surgery for the primary IGM	10 (71.43%)
Wide local re-excision	14 (100%)
Prednisolone treatment	14 (100%)

Data presented as frequency and percentages unless otherwise indicated. BMI, Body mass index.

**Table 3:** Correlation analysis of clinical characteristics of IGM with patient age and BMI at presentation.

Characteristics	All patients (*n* = 44)				Patients with recurrence (*n* = 14)			
	Age, years		BMI, kg/m^2^		Age, years		BMI, kg/m^2^	
	rho	*p*-value	rho	*p*-value	rho	*p*-value	rho	*p*-value
Size of mass at presentation, cm	0.038	0.862	−0.570	**0.007 ** ^∗^	−0.241	0.566	−0.879	**0.009 ** ^∗^
Duration for presence of mass, months	−0.049	0.809	0.230	0.280	−0.182	0.615	0.025	0.948
Duration of prednisolone treatment, weeks	0.326	0.327	0.350	0.322	0.754	0.084	0.543	0.266
Size of tissue removed, cm	−0.001	0.995	0.145	0.509	0.428	0.218	0.168	0.666
Ratio between mass size and removed tissue	0.467	0.092	−0.286	0.368	0.410	0.493	0.211	0.789
Time to recurrence, months	—	—	—	—	−0.836	**0.003 ** ^∗^	−0.232	0.548
Number of recurrence episodes	—	—	—	—	0.102	0.766	0.172	0.636
Size of recurrence mass, cm	—	—	—	—	−0.633	0.127	0.087	0.870

rho, Spearman's correlation coefficient. ^∗^Indicates statistical significance at *p* < 0.05. BMI, body mass index.

Bold values represent statistically significant findings in correlation analysis.

**Table 4:** Changing trends of idiopathic granulomatous mastitis (IGM) at King Abdulla University Hospital.

Characteristics	(1994–2002)^[14]^	(2002–2018)
Number of patients	24	44
Mean age, years	34.3 (range: 11–55)	37.85 (range: 25–52)
Clinical initial diagnosis of IGM	None	70.5%
Initial false impression of carcinoma	71%	29.54%
*Tissue diagnosis*		
True-cut biopsy	None	31 patients (70.45%)
Frozen section	None	11 patients (25.00%)
Fine needle aspiration cytology	17 (70.83%)	14 patients (31.82%)
Post wide local surgical excision	24 (100.00%)	2 patients (4.55%)
Mastectomy performed due to a false clinical, radiological, and cytological evaluation	1 patient	None
Curative surgical excision	24 patients	43 patients
Use of steroids	4 patients (16.67%)	19 patients (43.18%)

## Data Availability

No data were used to support this study.
